# Infarto Agudo do Miocárdio como Primeira Manifestação da Policitemia Vera

**DOI:** 10.36660/abc.20190104

**Published:** 2020-05-11

**Authors:** Caroline Ferreira da Silva Mazeto Pupo da Silveira, Lívia Beatriz Santos Limonta Vitali, Fabiana Garcia Faustino, Alejandra Del Carmen Villanueva Maurício, Renato Teixeira, Silméia Garcia Zanati Bazan

**Affiliations:** 1 Universidade Estadual Paulista Júlio de Mesquita Filho Faculdade de Medicina de Botucatu BotucatuSP Brasil Universidade Estadual Paulista Júlio de Mesquita Filho - Faculdade de Medicina de Botucatu (UNESP), Botucatu, SP - Brasil

**Keywords:** Infarto do Miocárdio, Policitemia Vera, Trombocitose, Fatores de Risco, Transtornos Mieloproliferativos

## Introdução

A policitemia vera (PV) é uma neoplasia mieloproliferativa clonal progressiva crônica caracterizada por um aumento absoluto nos eritrócitos e, geralmente, leucocitose, trombocitose e esplenomegalia. Sua incidência está em torno de 2,8/100.000 pessoas por ano.^1^ O diagnóstico é feito utilizando os critérios definidos pelas diretrizes revisadas da Organização Mundial da Saúde (OMS) de 2016.^2^ Os principais critérios são níveis de hemoglobina acima de 16,5 e 16,0 g/dL ou hematócritos acima de 49 e 48% em homens e mulheres, respectivamente, ou aumento da massa de glóbulos vermelhos em mais de 25% acima do valor médio normal previsto; biópsia da medula óssea apresentando hipercelularidade para a idade com crescimento de trilinhagem; presença de mutação no éxon 12 do gene JAK2V617F ou JAK2. Um critério menor é o nível sérico reduzido de eritropoietina. O diagnóstico requer que se atenda aos três critérios principais ou aos dois critérios principais e ao critério menor. O paciente também é considerado em risco de trombose; aqueles com mais de 60 anos ou com histórico de trombose são considerados de alto risco; se ambos os fatores de risco estiverem ausentes, é considerado baixo risco.

O tratamento inclui medicamentos citorredutores, como hidroxiureia, antiagregantes plaquetários e sangrias terapêuticas.

A trombose representa importante causa de morbidade e mortalidade em pacientes com PV. Esses eventos trombóticos são mais frequentemente microcirculatórios e arteriais.^2^

O infarto agudo do miocárdio (IAM) nas doenças mieloproliferativas é atribuído principalmente à trombose coronariana devido à hiperviscosidade e trombocitose. O risco aumenta na presença de fatores de risco cardiovascular.^3^ Os eventos coronarianos são comuns durante o acompanhamento da PV, com uma taxa de 11,4% no seguimento de 10 anos, conforme consta na literatura.^4 , 5^ Além disso, em estudos recentes, os eventos trombóticos arteriais foram mais comuns que os trombóticos venosos quando diagnosticados pouco antes do diagnóstico de PV. Entretanto, a primeira apresentação da PV como IAM é considerada rara, com menos de 10 casos na literatura.^3 , 6 - 15^

## Relato de caso

**Figura 1:**
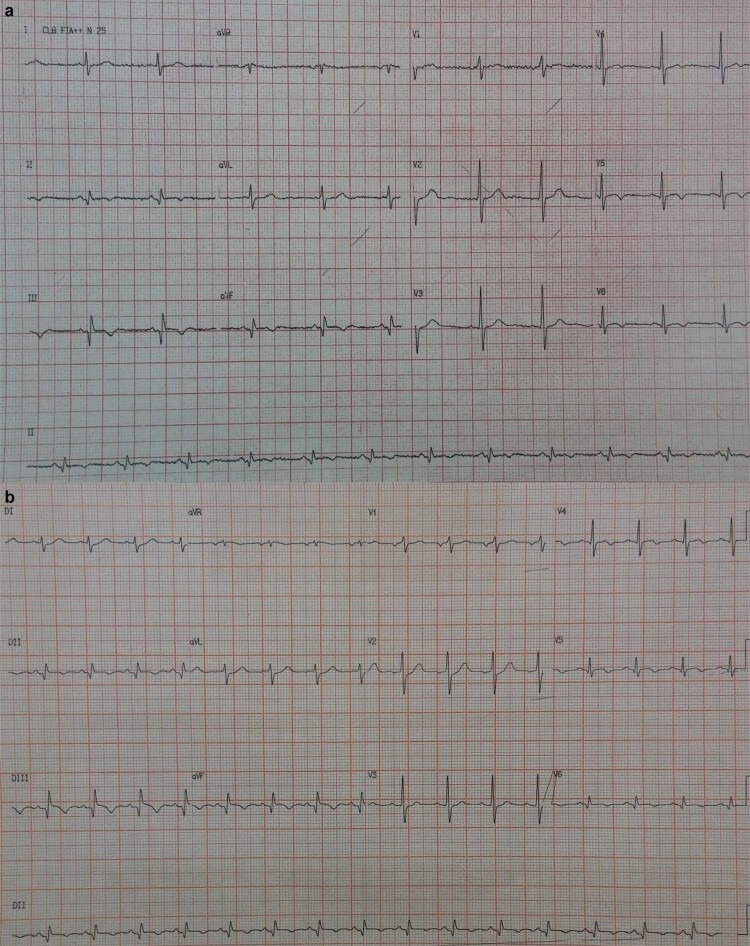
- Eletrocardiogramas: na admissão (a) com onda Q patológica, inversão da onda T em DII, DIII e aVF e inversão assimétrica da onda T nas derivações precordiais (V4–V6); e 1 hora após (b) com elevação do segmento ST em DII, DIII e aVF, mantendo as demais características.

**Figura 2:**
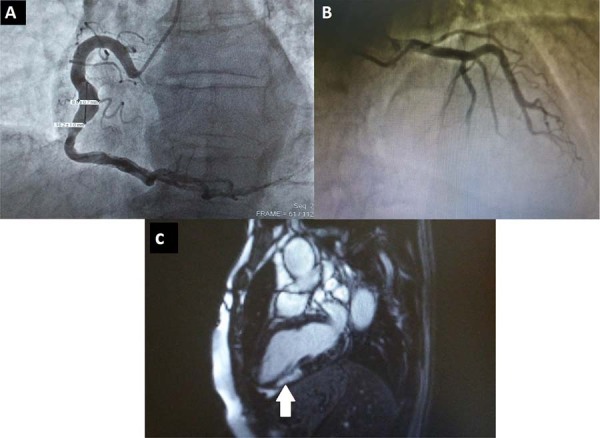
- A. Angiografia coronariana. Quadro mostrando a coronária direita com aneurisma, 8,0 mm de largura e 16,2 mm de comprimento; B. Angiografia coronária. Quadro mostrando o grau 3 de fluxo TIMI da artéria coronária descendente anterior esquerda; C. Ressonância magnética cardíaca. Quadro mostrando realce tardio de gadolínio no segmento apical inferior, padrão isquêmico.

Paciente do sexo masculino, 68 anos de idade, branco, em tratamento regular para hipertensão, sem histórico de eventos trombóticos. Deu entrada no serviço de emergência com mal-estar inespecífico, sem dor precordial ou dispneia, dormência na porção proximal de ambos os braços. Foi internado hemodinamicamente estável com boa saturação de oxigênio. No exame físico, apresentava face pletórica e macicez à percussão no espaço de Traube. Devido à probabilidade de apresentação atípica de síndrome coronariana aguda, inicialmente, o paciente foi investigado com eletrocardiograma (ECG) e marcadores de necrose miocárdica (MNM), hemograma completo e função renal. ECG em repouso ( Figura 1a ) mostrou onda Q patológica e inversão da onda T em DII, DIII e aVF, posteriormente evoluindo ( Figura 1b ) com elevação do segmento ST em DII, DIII e aVF, enquanto as demais características foram mantidas. Os MNM foram positivos (CK-MB de 34 a 36 ng/mL; referência <16 ng/dL e troponina de 0,12 a 0,81 e, depois, 1,07 ng/mL; referência <0,01 ng/mL). A embolia pulmonar foi descartada devido ao dímero D negativo. Outras análises laboratoriais mostraram função renal normal e hemoglobina 21,3 g/dL, hematócrito 65,4%, plaquetas 805.000/mm^3^ (referência: 140,000 - 440,000/mm^3^), caracterizando hiperviscosidade, macroplaquetas e leucócitos 15.400/mm^3^ (referência: 4,000–11,000/mm^3^) e, principalmente, neutrófilos. Também não mostrou alterações lipídicas ou de glicose. O paciente foi diagnosticado com IAM causado por PV e, ao contrário do que é mais encontrado na literatura, o diagnóstico de IAM ocorreu antes da descoberta da PV. Ele foi classificado como em alto risco de trombose devido à idade, tendo sido iniciada terapia antiplaquetária dupla com AAS (dose de ataque de 300 mg mais 100 mg/dia) e clopidogrel (dose de ataque de 300 mg mais 75 mg/dia), além de enoxaparina 1 mg/kg duas vezes ao dia. Como observado na Figura 1 , a elevação do segmento ST foi inferior a 1 mm. Além disso, os sintomas não pioraram com a alteração do ECG; portanto, a equipe optou por ponderar a relação benefício-risco em relação a submeter à angiografia um possível IAM com supradesnivelamento do segmento ST ou mesmo um IAM com supradesnivelamento do segmento ST com um nível elevado de hematócritos. O paciente foi então submetido a três sangrias terapêuticas antes que a angiografia coronária ( Figura 2A e 2B ) pudesse ser realizada com segurança, mostrando ausência de evidência angiográfica de trombo intracoronariano e dilatação aneurismática na porção mediana da artéria coronária direita e nenhuma alteração ou obstrução na artéria coronária descendente anterior esquerda ou artéria circunflexa. O paciente apresentava grau 3 de fluxo TIMI nas artérias coronária direita, circunflexa e descendente anterior esquerda ( Figura 2B ). Não há informações sobre a contagem de quadros TIMI. A avaliação fisiológica das artérias não estava disponível no serviço. Embora nenhum trombo tenha sido encontrado, pois pode ter sido causado pelo tratamento anterior à angiografia e, devido à ausência de outra hipótese, mantivemos o diagnóstico de IAM tipo 2. O ecocardiograma mostrou função sistólica preservada com fração de ejeção de 64% (Teichholz), disfunção diastólica leve (razão E/A de 1,0, razão E/e’ de 8,67) e nenhuma alteração da contratilidade ventricular esquerda. A área do IAM foi visualizada por ressonância magnética cardíaca ( Figura 2C ), que mostrou realce tardio do padrão isquêmico, compatível com o infarto definidor da área fibrótica da porção média e ápice da parede inferior, com fração de ejeção preservada. A ultrassonografia do abdome confirmou esplenomegalia homogênea e baixa eritropoietina (1,5 mUI/mL; referência 5,4–31,9 mUI/mL), e a mutação JAK-2 confirmou nossa hipótese. Iniciou-se tratamento com hidroxiureia, o clopidogrel foi suspenso e a anticoagulação foi mantida até a alta (8 dias). O paciente evoluiu sem complicações durante sua internação hospitalar ou durante o acompanhamento precoce.

## Discussão

Relatamos aqui um caso muito raro de primeira apresentação de PV como IAM. Até onde sabemos, menos de 10 casos semelhantes a esse foram relatados até o momento.^3^ Geralmente, os pacientes são diagnosticados com PV e, posteriormente, apresentam alguma forma de síndrome coronariana aguda, em cerca de 11,4% dos casos.^4^

Nosso paciente apresentava apenas hipertensão e idade como fatores de risco, e não apresentava alterações significativas no perfil lipídico, nível de glicemia de jejum, função renal ou histórico familiar que poderiam ter aumentado o risco de desenvolver IAM. No caso desse paciente, havia duas condições que poderiam ter contribuído para o infarto do miocárdio: o aneurisma coronariano e a própria PV, os quais podem contribuir para a formação de trombo e IAM.

Os mecanismos pelos quais a PV levaria a eventos vasculares ainda não estão bem estabelecidos. No entanto, algumas hipóteses foram apresentadas na literatura, como superprodução de tromboxano A2, disfunção endotelial e ativação plaquetária e de leucócitos.^16^ A elevação da contagem de leucócitos ocorre em 50 a 60% dos pacientes com PV, o que também pode ter um efeito prejudicial à microcirculação na PV. Os leucócitos ativados podem liberar proteases e radicais de oxigênio que alteram as células endoteliais e as plaquetas, a fim de favorecer o desenvolvimento de um estado protrombótico. Na PV, a quantidade de agregados plaquetas-leucócitos aumenta, estando associados ao aumento da propensão à trombose. Além disso, o estado protrombótico na PV tem sido atribuído a uma resistência adquirida ao anticoagulante natural — proteína C — que está associada a níveis reduzidos de proteína S^2^ . De acordo com essa afirmação, nosso paciente teve um aumento não apenas na contagem de plaquetas, mas também na contagem de leucócitos, principalmente neutrófilos, sem sinais de infecção, embora isso também possa corresponder ao processo inflamatório do IAM.

Outro achado interessante na literatura é que eventos trombóticos podem ocorrer mesmo quando os níveis de hematócritos e plaquetas são aceitáveis,^17^ indicando que o médico deve estar atento a esse diagnóstico diferencial, mesmo em doenças controladas.

Em conclusão, este é um caso raro de primeira manifestação de PV como IAM, curiosamente com a falta de obstrução na angiografia, indicando uma possível resolução do trombo após a terapia antiplaquetária. Nesses casos, o desafio continua sendo a terapia em pacientes com obstrução sustentada, uma vez que a colocação do stent pode significar um risco maior de oclusão subsequente devido à suscetibilidade do paciente a formar trombos plaquetários.
