# Supplementary material for: Infarto Agudo do Miocárdio como Primeira Manifestação da Policitemia Vera
Source: Arq Bras Cardiol. 2020 May 11;114(4 Suppl 1):27–30. [Article in Portuguese] doi: 10.36660/abc.20190104 (PMC8149117; doi:10.36660/abc.20190104)
Supplement: Translation

# Translation: Acute Myocardial Infarction as First Onset of Polycythemia Vera

**Authors:** Caroline Ferreira da Silva Mazeto Pupo da Silveira, Lívia Beatriz Santos Limonta Vitali, Fabiana Garcia Faustino, Alejandra Del Carmen Villanueva Maurício, Renato Teixeira, Silméia Garcia Zanati Bazan

**Affiliations:** 1 Brazil Universidade Estadual Paulista Júlio de Mesquita Filho - Faculdade de Medicina de Botucatu (UNESP), Botucatu, SP - Brazil

**Keywords:** Myocardial Infarction, Polycythemia Vera, Thrombocytosis, Myeloproliferative Disorders

## Introduction

Polycythemia vera (PV) is a chronic clonal progressive myeloproliferative neoplasm characterized by an absolute increase in erythrocytes and, usually, leukocytosis, thrombocytosis, and splenomegaly. Its incidence rates around 2.8/100,000 people per year.^1^ Diagnosis is confirmed using the criteria defined by the 2016 revised World Health Organization (WHO) guidelines.^2^ Major criteria include hemoglobin levels over 16.5 and 16.0 g/dL or hematocrit over 49 and 48% in men and women, respectively, or increased red cell mass of more than 25% above the mean normal predicted value; bone marrow biopsy showing hypercellularity for age with trilineage growth; presence of JAK2V617F or JAK2 exon 12 mutation. A minor criterion is reduced serum erythropoietin level. Diagnosis requires meeting either all 3 major criteria or 2 major criteria and the minor criterion. The patient is also considered as at thrombosis risk; those aged over 60 or with thrombosis history are considered at considered high risk; if both risk factors are absent, low risk is considered.

Treatment includes cytoreductive drugs, such as hydroxyurea, antiplatelet agents and therapeutic sangrias.

Thrombosis is a major cause of morbidity and mortality in PV patients. These thrombotic events are most frequently microcirculatory and arterial.^2^

Acute myocardial infarction (AMI) in myeloproliferative diseases is mostly attributed to coronary thrombosis due to hyperviscosity and thrombocytosis. The risk is increased in the presence of cardiovascular risk factors.^3^ Coronary events are common during the follow-up of PV, with a rate of 11.4% in 10-year follow-up in the literature.^4 , 5^ Also, in recent studies, arterial thrombotic events were more common than venous thrombotic events when diagnosed shortly before the PV diagnosis. However, the first presentation of PV as AMI is considered rare, with fewer than 10 cases in the literature.^3 , 6 - 15^

## Case Report

Patient was a 68 years-old white male, regularly treating hypertension, without any previous history of thrombotic events. He presented at the emergency room with an unspecific malaise, without chest pain or dyspnea, numbness in the proximal portion of both arms. He was admitted hemodynamically stable with good oxygen saturation. At physical examination, he had a plethoric face and dullness to percussion over Traube’s space. Due to the likelihood of atypical presentation of acute coronary syndrome, he was initially investigated with an electrocardiogram (ECG) and myocardial necrosis markers (MNM), blood cell count and kidney function. The rest ECG ( Figure 1a ) showed pathologic Q wave and inversion of T wave in DII, DIII and aVF, later evolving ( Figure 1b ) with elevation of the ST segment in DII, DIII and aVF, while the other characteristics were maintained. MNM came positive (CK-MB from 34 to 36 ng/mL; reference <16 ng/dL and troponin from 0.12 to 0.81 and then to 1.07 ng/mL; reference <0.01 ng/mL). Pulmonary embolism was ruled out due to negative D-dimer. Other laboratorial analysis showed normal renal function and hemoglobin 21.3 g/dL, hematocrit 65.4%, platelets 805,000/mm^3^(reference: 140,000–440,000/mm^3^), thus characterizing hyperviscosity, macroplatelets and leucocytes 15,400/mm^3^(reference: 4,000–11,000/mm^3^), mainly neutrophils. It also showed no lipids or glucose alterations. The patient was diagnosed with AMI caused by PV and, against what is mostly found in the literature, the AMI diagnosis came prior to the discovery of PV. He was classified as at high thrombosis risk due to his age and double anti-platelet therapy was initiated with AAS (loading dose of 300 mg plus 100 mg/day) and clopidogrel (loading dose of 300 mg plus 75 mg/day), as well as enoxaparin 1 mg/kg twice a day. As observed in Figure 1 , ST elevation was less than 1 mm. Also, the symptoms did not get worse as the ECG changed, so the team opted for weighing the benefit-risk ratio regarding submitting a possible non ST segment elevation AMI or even a non-reperfused ST segment elevation AMI to angiography under a high hematocrit situation. He was then submitted to 3 therapeutic sangrias before the coronary angiography ( Figure 2A and 2B ) could be performed safely, showing absence of angiographic evidence of intra-coronary thrombus and aneurysmatic dilatation in the median portion of the right coronary artery and no abnormalities or obstructions in the left anterior descending coronary artery or circumflex artery. The patient had TIMI 3 flow grade in the right coronary, circumflex and left anterior descending arteries ( Figure 2B ). There is no information on TIMI frame count. Physiology assessment of the arteries was not available in the service. Even though no thrombus was found, as this may have been caused by treatment prior to angiography, and due to the lack of another hypothesis, we sustained the diagnosis of type 2 AMI. Echocardiogram showed preserved systolic function with an ejection fraction of 64% (Teichholz), mild diastolic dysfunction (E/A ratio of 1.0, E/e’ ratio of 8.67) and no alteration of left ventricular contractility. The AMI area was viewed on cardiac magnetic resonance imaging ( Figure 2C ), which showed late enhancement of ischemic pattern, compatible with fibrotic area defining infarction of the medium and apex portion of the inferior wall, with preserved ejection fraction. Abdomen ultrasound confirmed homogeneous splenomegaly and low erythropoietin (1,5 mUI/mL; reference 5.4–31.9 mUI/mL), and JAK-2 mutation confirmed our hypothesis. He was then started on hydroxyurea, clopidogrel was suspended and anticoagulation was kept until discharge (8 days). The patient evolved without complications during his in-hospital stay or during early follow-up.

**Figure 1:** - Electrocardiograms: at admission (a) with pathologic Q wave, inversion of T wave in DII, DIII and aVF, and asymmetric inversion of T wave in the precordial leads (V4–V6); and 1 hour after (b) with ST segment elevation in DII, DIII and aVF, keeping the other characteristics.

**Figure 2:** - A. Coronary angiography. Frame showing the right coronary with aneurysm, 8.0-mm wide and 16.2-mm long; B. Coronary angiography. Frame showing the left anterior descending coronary artery TIMI 3 flow; C. Cardiac magnetic resonance imaging. Frame showing late gadolinium enhancement in the apical inferior segment, ischemic pattern.

## Discussion

We report here a very rare case of first presentation of PV as AMI. To our knowledge, fewer than 10 cases similar to this have been reported so far.^3^ Usually, the patients are diagnosed with PV and, later, present some form of coronary acute syndrome, in about 11.4% of cases.^4^

Our patient had only hypertension and age as risk factors, and had no significant alterations in lipid profile, fasting glucose level, renal function or family history that could have increased the risk of developing AMI. In this patient’s case, there were two conditions that could have contributed to myocardial infarction: the coronary aneurysm and the PV itself, both of which can contribute to the formation of thrombus and AMI.

The mechanisms through which PV would lead to vascular events are not yet well established. However, some hypotheses have been displayed in literature, such as overproduction of thromboxane A2, endothelial dysfunction and platelet and leukocyte activation.^16^ Elevation of leukocyte count occurs in 50 to 60% of PV patients, which may also have a detrimental effect on microcirculation in PV. Activated leukocytes may release proteases and oxygen radicals that alter endothelial cells and platelets in order to favor the development of a prothrombotic state. Platelet-leukocyte aggregates are increased in number in PV and are associated with increased propensity of thrombosis. In addition, the prothrombotic state in PV has been attributed to an acquired resistance to the naturally occurring anticoagulant — protein C — which is associated with reduced levels of protein S^2^ . In agreement with that statement, our patient had an increase not only in platelet but also in leukocyte count, mainly neutrophils, without any signs of infection, although that could also correspond to the AMI inflammation process.

Another interesting finding in the literature is that thrombotic events might happen even when hematocrit and platelet levels are acceptable^, 17^ indicating that the physician should be alert to that differential diagnosis even in controlled diseases.

In conclusion, this is a rare case of first onset of PV as AMI, interestingly with lack of obstruction in the angiography, indicating a possible resolution of the thrombus after antiplatelet therapy. The challenge in those cases remains the therapy in patients with sustained obstruction, once stent placing might mean a higher risk of subsequent occlusion due to the patient’s susceptibility to form platelet thrombi.
